# Heat Source Characteristics of Ternary-Gas-Shielded Tandem Narrow-Gap GMAW

**DOI:** 10.3390/ma12091397

**Published:** 2019-04-29

**Authors:** Xiaoyu Cai, Bolun Dong, Sanbao Lin, Anthony B. Murphy, Chenglei Fan, Chunli Yang

**Affiliations:** 1 State Key Laboratory of Advanced Welding and Joining, Harbin Institute of Technology, Harbin 150001, China; xycai@hit.edu.cn (X.C.); dongbolun@hotmail.com (B.D.); fclwh@hit.edu.cn (C.F.); yangcl9@hit.edu.cn (C.Y.); 2 CSIRO Manufacturing, P.O. Box 218, Lindfield NSW 2070, Australia; Tony.Murphy@csiro.au

**Keywords:** narrow-gap welding, shielding gas, helium, heat source

## Abstract

The characteristics of the welding heat source for tandem narrow-gap gas metal arc welding are examined for different ternary shielding gas (Ar-CO_2_-He) compositions. Results of previous calculations of arc properties for bead-on-plate geometry are adapted to the narrow-gap geometry to predict these characteristics. The heat source concentration factor decreases and the maximum heat flux density increases as the helium content increases, which leads to an increased welding heat efficiency. Addition of CO_2_ up to around 10% also increases the heat efficiency. When the CO_2_ content exceeds 10%, the heat source concentration factor increases significantly and the heat efficiency decreases. The shielding gas composition also affects the heat source distribution. The heat source characteristics are applied to a computational fluid dynamic model of the weld pool to predict the weld shape, and the predictions are verified by experiment. The results indicate that the appropriate addition of helium to the shielding gas can increase the heat transferred to the peripheral regions of the arc and increase the sidewall penetration.

## 1. Introduction

Narrow-gap (NG) welding is an efficient welding method for thick plates [1], in which a narrow and deep gap is used instead of wide-angle groove. Narrow gap arc welding contains narrow gap tungsten arc welding (NG-GTAW), narrow gap gas metal arc welding (NG-GMAW) and narrow gap submerged arc welding (NG-SAW). Compared with NG-GTAW and NG-SAW, NG-GMAW has a high welding efficiency; heat input is low and the welded joint has good properties, so the NG-GMAW has a better application prospect. However, the lack of sidewall fusion is the most frequent defect in GMAW [2]. The shielding gas composition can modify the arc properties and heat transfer performance, which will affect the final weld formation.

Helium has special physical properties, such as a higher thermal conductivity and a higher ionization energy. Therefore, it is expected that more heat can be transferred to the peripheral regions of the arc, which leads to the increase of the metal fusion area. Urmston [3] found that a more rounded weld profile could be obtained with the addition of helium to the shielding gas in GMAW. Thompson [4] pointed out that the weld lateral fusion increased by 60% when the helium content increased to 5% in pulsed GMAW. In previous studies [5], it was found that in narrow-gap GMAW, the addition of helium can increase the sidewall penetration by up to 40%. However, the effects of the shielding gas on the arc properties, droplet transfer and the weld formation were discussed only through experiments. Due to the deep and narrow groove in narrow-gap welding, it is difficult to observe the molten pool behavior experimentally. With the development of computational methods and technology, numerical simulation has become a widely used tool in welding process development and optimization. There have been several previous efforts to evaluate the molten pool flow in narrow-gap GMAW by numerical simulation. In our previous study [6], arc properties with different helium contents were investigated using numerical simulation for a standard bead-on-plate GMAW configuration. The results indicated that the helium content led to changes to the arc properties that will affect the heat transfer. 

Using these results, in this study the characteristics of the heat source for narrow-gap welding are investigated using geometric considerations, and heat source models for different shielding gas compositions are developed. The heat source models are then applied to a computational fluid dynamic model of the weld pool. This approach is much simpler than developing a fully coupled simulation of the arc and weld pool, but nevertheless provides important insights into the influence of different shielding gas mixtures while giving predictions in accordance with measurements of weld cross-sections. The effects of shielding gas composition on the heat source characteristics are analyzed, and the mechanisms by which the shielding gas influences the molten pool temperature field and weld formation are studied. The results of this study can provide theoretical foundation to the application of the Ar–CO_2_–He ternary shielding gas in narrow-gap GMAW.

## 2. Methods

### 2.1. Experimental Setup

**Figure 1:**
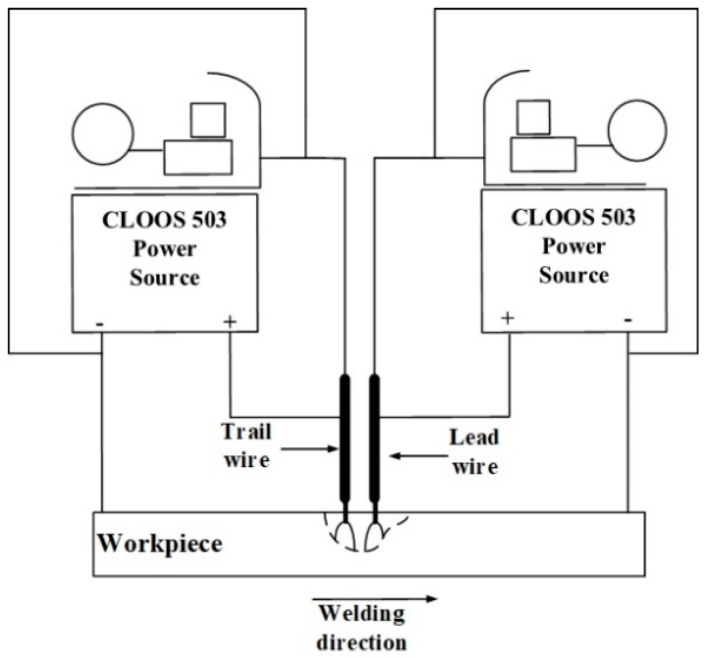
Experimental apparatus.

Figure 1 shows the experimental apparatus. The two CLOOS 503 power sources (CLOOS, Haiger, Germany) controlled the two wires independently. The distance between the two wires was 15 mm, and the two arcs attach to a single molten pool. The power sources were operated in pulsed mode, in which the peak voltage and the base current were both held constant. 

**Figure 2:**
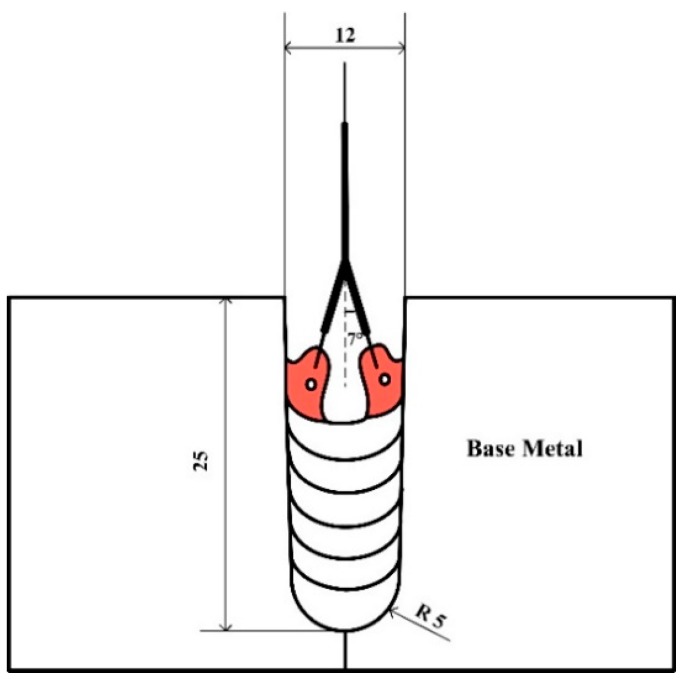
Geometrical size of the groove.

The geometric dimensions are presented in Figure 2, the ends of the two contact tips were bent to direct the two wires toward the opposite sidewalls to ensure sufficient sidewall penetration. 

Q235 was the base metal and H08Mn2Si with the diameter of 1.2 mm was the filler metal. The welding parameters are given in Table 1. The design of the shielding gas compositions is shown in Table 2. In order to highlight the effects of He or CO_2_, each component (CO_2_/He) was varied within a range while the other component (He/CO_2_) remained constant. The percentages in the gas composition are vol%.

### 2.2. Computational Model

Numerical simulation was used to calculate the molten pool temperature and modify the heat source model, in order to understand the effects of shielding gas on the welding heat source. Some assumptions were made as follows:

(1) The molten metal is an incompressible Newtonian fluid.
(2) The flow of the fluid is laminar.
(3) The droplet is spherical and transfers at a constant speed.

On the basis of the above assumptions, numerical simulation was used to study the heat source. The governing equations, the boundary conditions and the source terms used are as given in a previous paper [7]. The main material physical properties are given in Table 3. Especially, the specific heat and thermal conductivity changes with the temperature, and the values are presented in Table 4.

**Figure 3:**
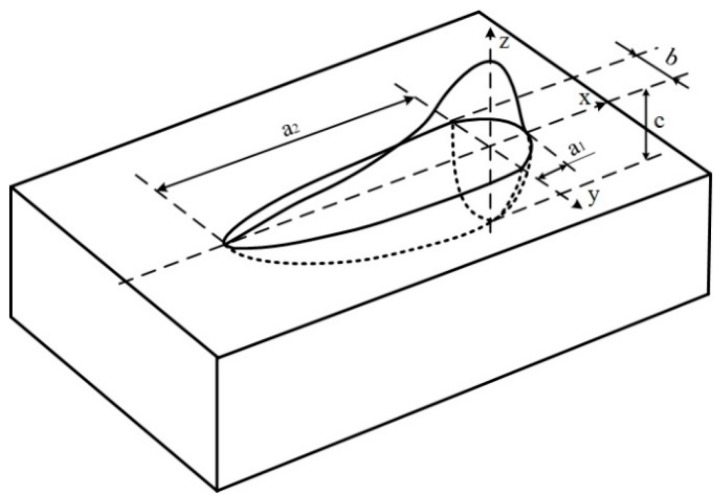
Double-ellipsoid heat source model.

As shown in Figure 3, a double-ellipsoid heat source was employed in this simulation model; the heat flux distribution are given as Equations (1)–(3).

Heat source distribution in the front half of the model [8]:

(1) $$q(x,y,z)=\eta_{h}UI\frac{6\sqrt{3}f_{1}}{\pi a_{1}bc\sqrt{\pi }}\exp(-3\frac{x^{2}}{a_{1}^{2}}-3\frac{y^{2}}{b^{2}}-3\frac{z^{2}}{c^{2}})$$

Heat source distribution in the back half of the model [8]:

(2) $$q(x,y,z)=\eta_{h}UI\frac{6\sqrt{3}f_{2}}{\pi a_{2}bc\sqrt{\pi }}\exp(-3\frac{x^{2}}{a_{2}^{2}}-3\frac{y^{2}}{b^{2}}-3\frac{z^{2}}{c^{2}})$$

(3) $$f_{1}+f_{2}=2$$

where *η* is the heating efficiency; *a*, *b*, *c* are geometrical parameters; *U* is the welding voltage; *I* is the welding current; *f* is the energy distribution coefficients of two halves of the heat source; *f*_1_ = 0.4 and *f*_2_ = 1.6.

**Figure 4:**
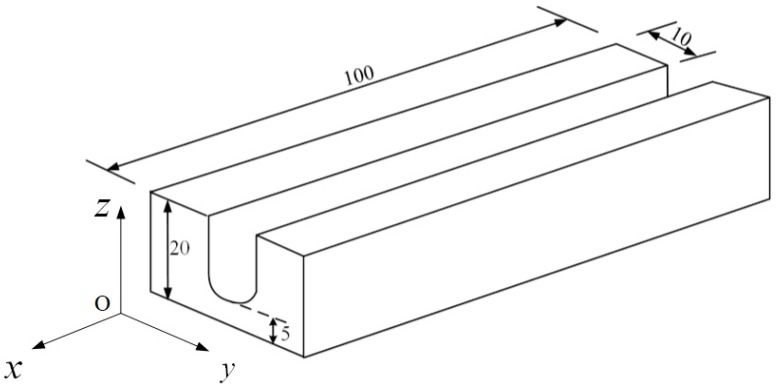
3D computational domain.

The 3D computational domain is shown in Figure 4. The bottom width of the gap is 10 mm, the top width is 12 mm, and the depth is 25 mm, as given in Section 2.1.

## 3. Results and Discussion

### 3.1. Heat Source Characteristics for Narrow-Gap Welding

**Figure 5:**
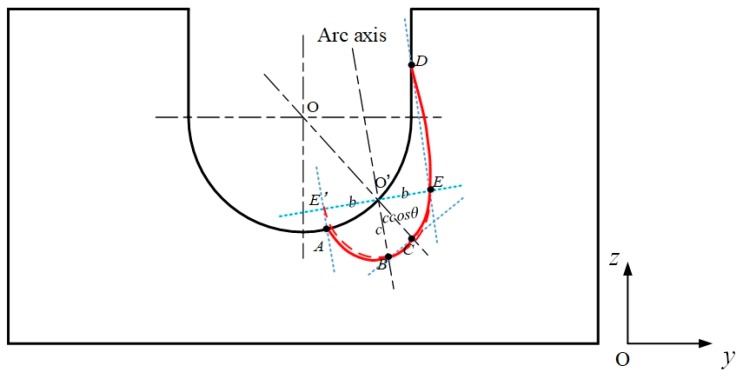
Heat distribution in narrow-gap welding.

The wires are directed towards the sidewalls, and the groove is narrow and deep. Therefore, the heat source is different from that of the bead-on-plate welding. As shown in Figure 5, *O’B* is the arc axis, *EE’* is the surface of the plate. *E*, *E’* and *B* are the points where the heat flux decreases to 0.05q*_max_ for bead-on-plate welding. The length of *O’E* and *O’E’* is the *b* value, and the length of *O’B* is the *c* value. The groove is uniform in the *x* direction, so the value of *a* is independent of the orientation of the arc axis, which is in the *yOz* plane. However, the values of *b* and *c* will change with the arc axis orientation. A line is drawn passing through *E* and line parallel to the arc axis. The line and the sidewall meet at *D*, which is the point at which the heat flux decreases to 0.05q*_max_ for narrow-gap welding. In the same way, on the surface of the groove bottom, *A* is the also the point where the heat flux decreases to 0.05q*_max_. If the line *EE’* is rotated on the axis of *O’* so that it is tangential to the groove surface, then *C* is the point at which the heat flux decreases to 0.05q*_max_. The length of *O’C* is *c·cosθ*. So in the *yOz* plane, the area bounded by the curve *A-B-C-E-D-A* is that in which the arc heat flux is distributed. It can be seen that, in narrow-gap welding, the heat distribution can be divided into two parts: One mainly distributed in the sidewall (*CEDO’C*) to melt the sidewall; the other (*ABCO’A*) distributed in the groove bottom to ensure the penetration depth. Using on the geometric relationships, it can be found that a great proportion of the heat will be transferred into the sidewall as *b* or *c* increases. 

### 3.2. Effects of Shielding Gas on Heat Source

**Figure 6:**
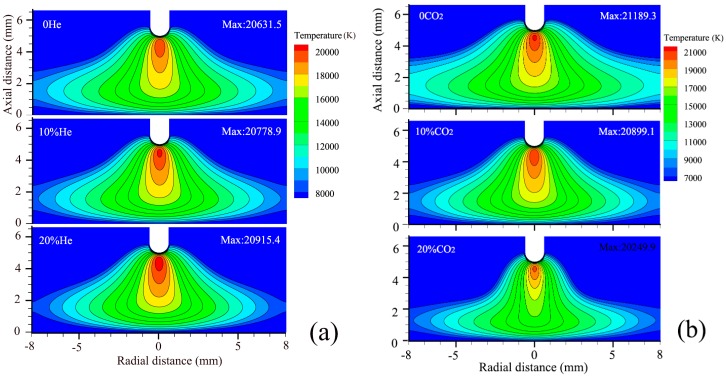
Arc temperature distribution under different helium (**a**) or CO_2_ contents (**b**) for bead-on-plate geometry; the CO_2_ content is 10% in (**a**) and the He content is 5% in (**b**).

**Figure 7:**
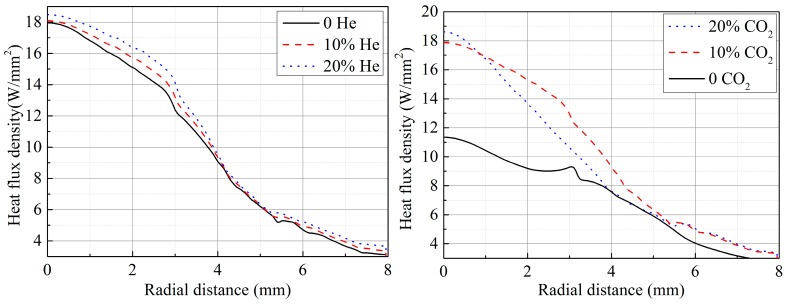
Heat flux density distribution for different helium or CO_2_ contents.

In our previous study [6], the arc properties were calculated. The arc temperature distribution and the heat flux density on the plate surface were obtained, as shown in Figure 6 and Figure 7 [6], respectively. 

It can be seen that, as the helium content increases, the heat flux density increases, which means that the welding heat efficiency increases. In this study, a double-ellipsoid heat source model was used, and it was transformed from the moving Gaussian heat source model. The main parameters that affect the double-ellipsoid properties are the welding heat efficiency, the welding parameters and the heat source model geometrical parameters (*a*, *b*, *c*). The shielding gas composition will affect the welding heat efficiency and the heat source model geometrical parameters.

In the *yOz* plane, the heat source behaves as a Gaussian heat source. In this case, the heat flux density distribution follows the following equation [9].

(4) $$q^{*}=q^{*}{}_{max}\exp(-kr^{2})$$

where $q^{*}$ is the heat flux density, $q^{*}{}_{max}$ is the maximum heat flux density, *k* is the heat concentration factor, and *r* is the radial distance from the arc axis. The heating power *q* can be obtained by integration.

(5) $$q=\int_{A}q^{*}(r)dA=\int_{0}^{\infty }q_{max}^{*}exp(-kr^{{}_{2}})\cdot 2\pi r\cdot dr=\frac{\pi }{k}q_{max}^{*}$$

where *A* is the area covered by the arc.

In arc welding, the heating power can be expressed in terms of the electrical energy [9]:

(6) $$q=\eta_{h}UI$$

The heat efficiency can be obtained from Equations (5) and (6).

(7) $$\eta_{h}=\frac{\pi q_{max}^{*}}{kUI}$$

It can be seen that, under the same welding parameters, the value of *η_h_* is related to *k* and q^*^_max_. As shown in Figure 7, as the helium or CO_2_ content increases, the value of q^*^_max_ increases. The heat flux density $q^{*}$ increases with the helium content, because of two main effects. First, the higher helium content decreases the electrical conductivity at temperatures below 20,000 K, causing current to flow closer to the arc axis and leads to a more constricted central region of the arc with a higher temperature [10]. Second, the thermal conductivity is increased, leading to better conductive heat transfer from the arc to the plate. The increased thermal conductivity leads to an expansion of the arc and furthermore the conductive heat transfer to the plate is increased at all radii, so the value of *k* decreases slightly. The welding heat efficiency therefore increases as the helium content increases according to Equation (7). 

Carbon dioxide is a triatomic gas, which dissociates in the upper part of the arc and re-combines near the weld pool surface. The dissociation and recombination reactions lead to large peaks in the specific heat and thermal conductivity at the dissociation temperatures, around 3500 K for the reactions

(8) $$2{\mathrm{CO}}_{2}\Leftrightarrow 2\mathrm{CO}+O_{2}$$

and

(9) $$O_{2}\Leftrightarrow 2O$$

and around 7000 K for the reaction

(10) CO⇔C+O

The increased specific heat c_p_ leads to a higher volumetric enthalpy $h\left(T\right)=\rho \left(T\right)\int_{300\;K}^{T}c_{p}\left(T\right)dT$, where *ρ* is the mass density, which in turn leads to a more constricted arc through the thermal pinch effect [11]. The energy required for the dissociation of CO_2_ leads to a decrease in arc temperature if the power is held constant. The peaks in thermal conductivity at the dissociation temperatures lead to an increased conductive heat transfer to the plate. An appropriate increase of the CO_2_ content can increase both $q^{*}$ and *η_h_*, but if the CO_2_ content is large (>20%), the arc temperature decreases more and the arc constricts strongly, and more heat is concentrated around the arc axis. Therefore, although $q^{*}{}_{max}$ increases as the CO_2_ content increases, *k* also increases. The substantial increase of the *k* value makes the *η_h_* decrease in spite of the increase of $q^{*}{}_{max}$.

The geometrical parameters are also influenced by the shielding gas composition. As the helium content increases, the heat flux density increases, so *a, b* and *c* should all increase in theory. In the same way, with increasing of the CO_2_ content, *c* increases and *a* and *b* increase initially and then decrease. However, in the numerical simulation of arc properties, the arc length was assumed to be constant for the different shielding gases, while in the actual GMA welding process, the arc length decreases as the helium or CO_2_ content increases to keep the voltage constant, as was discussed in detail in our previous work [12]. The arc length decrease reduces the arc root area and contributes to a decrease in *a* and *b*. Therefore, the heat transfer characteristics of the arc and the arc length work together to affect the heat source geometrical parameters.

### 3.3. Heat Sources under Different Shielding Gases

**Figure 8:**
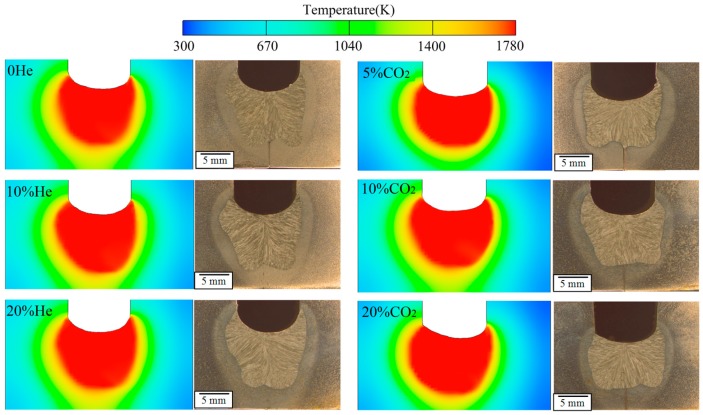
Calculated molten pool temperature fields and the corresponding measured weld shapes; the CO_2_ content is 10% in the left-hand figures and the He content is 5% in the right-hand figures.

Based on the above discussion, the heat source models for different shielding compositions were modified according to the weld shape. The predictions of the model using the heat source models are given in Figure 8, with the measured weld cross-section also shown. In the images, the left is the side of the lead wire and the right is the side of the trail wire. It can be seen that the fusion line of the molten pool and the fusion line of the weld are in good agreement. The weld shape in tandem with narrow-gap welding is asymmetrical. In the welding process, the lead arc heats the groove bottom surface directly, and when the molten pool on the side of lead wire forms, the molten metal flows to the trail edge of the pool. Hence, the trail arc works on the surface of the molten metal, so the depth of sidewall penetration of this side is shallower than the other side because of the cushion effect. The weld width increases with the increase of CO_2_ content (0–10%). When the CO_2_ content exceeds 10%, the arc length clearly decreases as the CO_2_ content increases [13], causing a decrease in the area of the arc root that acts on the sidewall, so the heat model geometrical parameters (*a* and *b*) decrease and the weld width decreases. Similarly, as the helium content increases, the arc length decreases and the weld width decreases when the helium content reaches 20%.

The heat source parameters used are given in Table 5. It can be seen that, as the helium content increases, the welding heat efficiency increases. The heat model geometrical parameters change: *a* and *b* initially increase and then decrease with increasing helium or CO_2_ content. The geometrical parameters and weld width are greatest when the helium and CO_2_ content are both 10%.

The heat source parameters are determined by both the heat transfer characteristics of the arc and the arc length. As the helium content increases, the heat efficiency increases but the arc length decreases. The decreased arc length leads to lower values of *a* and *b*, as discussed above. As the CO_2_ content increases, the heat efficiency initially increases and then decreases due to the effects of the CO_2_ decomposition. Because the arc length decreases sharply as the CO_2_ content increases, the geometrical parameters decrease further when the CO_2_ content reaches 20%.

## 4. Conclusions

The heat source distribution in narrow-gap welding is different from that in bead-on-plate welding. One part of the heat source is distributed on the sidewall where it melts the sidewall metal, and the other part is distributed on the groove bottom to ensure depth of penetration. Changing the heat source affects the melting behavior and hence the weld formation.

The shielding gas composition affects the arc properties and therefore the heat source characteristics. As the helium content increases, the heat concentration factor decreases, which leads to an increased heat efficiency. Large CO_2_ content causes an increase in the heat concentration factor, which leads to a decrease in the heat efficiency in spite of an increased maximum heat flux density.

The geometrical parameters of the heat source model are also influenced by the shielding gas composition. With increasing helium content, more heat is transferred to the peripheral region of the arc but the arc length decreases, with the consequence that the geometrical parameters initially increase and then decrease. When the CO_2_ content increases to 20%, the arc constricts and the arc length decreases substantially, so the geometrical parameters also initially increase and then decrease with increasing CO_2_ content.

The approach developed here, in which a simulation of a single arc in a simple welding geometry is used to provide an initial heat source distribution, and the adaptation of the parameters of this heat source distribution to a more complex geometry and a tandem arc, is found to be an efficient and effective for predicting weld profiles. The approach is readily applicable to other weld geometries and shielding gas mixtures.

## 5. Perspectives

In the narrow-gap welding, the sidewall penetration increases with the addition of the helium. Helium has a good application potential in narrow-gap welding. Some studies can be done in the future.

(1) The narrow-gap welding arc properties under different ternary shielding gas compositions can be studied by numerical simulation.
(2) The effects of the gas mixtures compositions on the weld microstructure and mechanical properties is a valuable research issue.

## Figures and Tables

**Table 1:** Welding parameters.

Wire Feed Speed (Lead/Trail) (m/min)	Pulse Frequency (Hz)	Pulse Period (ms)	Peak Voltage (V)	Base Current (A)	Welding Speed (mm/min)
10/10	220	2.0	34	60	300

**Table 2:** Design of the shielding gas compositions.

Weld	Ar (%)	CO_2_ (%)	He (%)
1	90	10	0
2	80	10	10
3	70	10	20
4	90	5	5
5	85	10	5
6	75	20	5

**Table 3:** Material physical properties employed in the simulation [7].

Nomenclature		Value	Nomenclature		Value
Solid density	ρ_s_/(kg m^−3^)	7990	Liquidus temperature	T_L_/(°C)	1460
Liquid density	ρ_l_/(kg m^−3^)	7200	Solidus temperature	T_S_/(°C)	1413
Temperature coefficient of surface tension	dσ/dT /(N m^−1^ °C^−1^)	−0.00035	Radiation emissivity	ε	0.8
Latent heat of fusion	H_f_/(J kg^−1^)	2.75 × 10^5^	heat transfer coefficient	h_conv_/(W m^−2^ K^−1^)	100
Viscosity	μ/(Pa S)	0.006	Room temperature	T_0_/(°C)	25
Surface tension	σ/(N m^−1^)	1.8	Permeability of vacuum	μ_0_/(B H^−1^)	1.2566 × 10^−6^
Wetting angle	θ/(°)	15			

**Table 4:** Specific heat and thermal conductivity with elevated temperature.

Temperature (°C)	20	250	500	800	1000	1500	1700	2500
Specific Heat (J kg^−1^ K^−1^)	460	480	530	675	670	660	780	820
Thermal Conductivity (W m^−1^ K^−1^)	50	47	40	26	28	50	140	142

**Table 5:** Heat source model geometry under different shielding gases.

Gas Mixer	*η_h_*	*a*_1_ (mm)	*a*_2_ (mm)	*b* (mm)	*c* (mm)
90%Ar-10%CO_2_	0.5	3	4	3	3
80%Ar-10%CO_2_-10%He	0.6	3.3	4.5	3.3	3.2
70%Ar-10%CO_2_-20%He	0.65	2.8	3.6	2.8	2.9
90%Ar-5%CO_2_-5%He	0.52	2.6	3.3	2.6	2.8
85%Ar-10%CO_2_-5%He	0.55	3.1	4.2	3.1	3.1
75%Ar-20%CO_2_-5%He	0.54	2.4	3	2.5	3.2
